# The Impact of ^68^Gallium DOTA PET/CT in Managing Patients With Sporadic and Familial Pancreatic Neuroendocrine Tumours

**DOI:** 10.3389/fendo.2021.654975

**Published:** 2021-06-07

**Authors:** Daniel J. Cuthbertson, Jorge Barriuso, Angela Lamarca, Prakash Manoharan, Thomas Westwood, Matthew Jaffa, Stephen W. Fenwick, Christina Nuttall, Fiona Lalloo, Andreas Prachalias, Michail Pizanias, Hulya Wieshmann, Mairead G. McNamara, Richard Hubner, Raj Srirajaskanthan, Gillian Vivian, John Ramage, Martin O. Weickert, D Mark Pritchard, Sobhan Vinjamuri, Juan Valle, Vincent S. Yip

**Affiliations:** ^1^ Liverpool University Hospitals NHS Foundation Trust, ENETS Centre of Excellence, Liverpool, United Kingdom; ^2^ Faculty of Health and Life Sciences, University of Liverpool, Liverpool, United Kingdom; ^3^ Division of Cancer Sciences, University of Manchester, Manchester, United Kingdom; ^4^ Department of Medical Oncology, The Christie NHS Foundation Trust, ENETS Centre of Excellence, Manchester, United Kingdom; ^5^ Department of Radiology and Nuclear Medicine, The Christie NHS Foundation Trust ENETS Centre of Excellence, Manchester, United Kingdom; ^6^ Department of Clinical Genetics, Manchester Centre for Genomic Medicine, Central Manchester University Hospitals NHS Foundation Trust, Saint Mary’s Hospital, Manchester, United Kingdom; ^7^ Institute of Liver Studies, Kings College Hospital, London, United Kingdom; ^8^ Neuroendocrine Tumour Unit, KHP ENETS Centre of Excellence, Institute of Liver Studies, Kings College Hospital, London, United Kingdom; ^9^ The Arden Neuroendocrine Centre, ENETS Centre of Excellence, University Hospitals Coventry and Warwickshire, Coventry, United Kingdom; ^10^ Barts and the London HPB Centre, Royal London Hospital, London, United Kingdom; ^11^ Department of Pancreatobiliary Surgery, Royal Liverpool University Hospital, Liverpool, United Kingdom

**Keywords:** pancreatic neuroendocrine tumours, ^68^Gallium DOTA PET/CT, functional imaging, multiple endocrine neoplasia type 1, sporadic and familial

## Abstract

**Objective:**

Pancreatic neuroendocrine tumours (panNETs) arise sporadically or as part of a genetic predisposition syndrome. CT/MRI, endoscopic ultrasonography and functional imaging using Octreoscan localise and stage disease. This study aimed to evaluate the complementary role of ^68^Gallium (^68^Ga)-DOTA PET/CT in managing patients with panNETs.

**Design:**

A retrospective study conducted across three tertiary UK NET referral centres.

**Methods:**

Demographic, clinical, biochemical, cross-sectional and functional imaging data were collected from patients who had undergone a ^68^Ga-DOTA PET/CT scan for a suspected panNET.

**Results:**

We collected data for 183 patients (97 male): median (SD) age 63 (14.9) years, 89.1 *vs.* 9.3% (n=163 vs. 17) alive *vs.* dead (3 data missing), 141 sporadic *vs.* 42 familial (MEN1, n=36; 85.7%) panNETs. Non-functional *vs.* functional tumours comprised 73.2 *vs.* 21.3% (n=134 *vs.* 39) (10 missing). Histological confirmation was available in 89% of individuals (n=163) but tumour grading (Ki67 classiifcation) was technically possible only in a smaller cohort (n=143): grade 1, 50.3% (n=72); grade 2, 46.2% (n=66) and grade 3, 3.5% (n=5) (40 histopathological classification either not technically feasible or biopsy not perfomed). 60.1% (n=110) were localised, 14.2% (n=26) locally advanced and 23.5% (n=43) metastatic (4 missing). 224 ^68^Ga-DOTA PET/CT scans were performed in total for: diagnosis/staging 40% (n=88), post-operative assessment/clinical surveillance 53% (n=117) and consideration of peptide receptor radionuclide therapy (PRRT) 8% (n=17) (2 missing). PET/CT results confirmed other imaging findings (53%), identified new disease sites (28.5%) and excluded suspected disease (5%). Overall, ^68^Ga-DOTA PET/CT imaging findings provided additional information in 119 (54%) patients and influenced management in 85 (39%) cases.

**Conclusion:**

^68^Ga-DOTA PET/CT imaging more accurately stages and guides treatment in patients with sporadic/familial panNETs with newly diagnosed/recurrent disease.

## Introduction

Pancreatic neuroendocrine tumours (panNETs) are rare tumours accounting for ~2% of all pancreatic malignancies (1). However, national registry data suggest there is an increasing incidence of NETs (up to 2.5-fold higher) across all sites, stages and grade of disease (overall annual age-adjusted incidence rate of NETs ~7/100,000 US population; and panNETs specifically 0.48/100,000) (2). NET prevalence is considerably higher, with more patients presenting with earlier stage disease (3), better overall survival (4) and a variety of systemic treatments for grade (G), G1/G2 tumours that prolong progression-free or overall survival, including somatostatin analogues, ^177^Lu-DOTATATE, systemic chemotherapy, everolimus and sunitinib (5–10).

An updated World Health Organisation (WHO) grading classification in 2017 suggested that pancreatic neuroendocrine neoplasms (p-NENs) be categorised according to the grade and degree of differentiation into *well-differentiated* pancreatic neuroendocrine tumours (panNETs) and *poorly differentiated* neuroendocrine carcinomas (NEC) (small cell and large cell subtypes) (11–13). Pancreatic NETs may be classified according to stage/disease extent (localised, regional or metastatic) and according to the histological grade using the Ki67 proliferation index from slower growing G1 and G2 tumours (G1/G2; Ki67 index <3% and 3-20% respectively) through to faster growing (well differentiated) G3 tumours (Ki67 index >20%) (14). Pancreatic NECs are characterised by a higher proliferative index (Ki67>20%) with usually a much more aggressive course (as for other poorly differentiated tumours). Histopathological data has validated prognostic value, independently predicting overall survival (15).

PanNETs may also be classified according to functionality, depending on secretion of hormones/peptides that cause specific symptoms/clinical syndromes. Patients with non-functional tumours, constituting the majority, tend to present late in the disease, often with distant metastases, and may have shorter life expectancy than those with functional panNETs (16). Finally, panNETs may arise sporadically or as part of a cancer-predisposing syndrome, such as in multiple endocrine neoplasia Type 1 (MEN1), von Hippel Lindau syndrome (VHL), tuberous sclerosis or neurofibromatosis (17). Functional panNETs occur more commonly in familial *vs.* sporadic panNETs (~40 *vs*. 10%); most commonly, insulinomas or gastrinomas (4). Indeed, panNETs occur in approximately two thirds of MEN1 patients and account for ~10% of all patients with panNETs (18).

Treatment of panNETs is dependent on multiple factors including tumour size, functionality, histological grade and stage. Imaging techniques used to stage NETs involve a combination of cross-sectional imaging (computer tomography [CT]/magnetic resonance imaging [MRI]) and endoscopic ultrasound (EUS). NETs generally over-express cell-surface somatostatin receptors (SSTRs), particularly subtypes 2a and 5, forming the basis for their detection and treatment using peptide receptor radionuclide therapy (PRRT) (19). In most NET specialist centres, functional imaging for staging of G1/G2 NETs involves somatostatin receptor scintigraphy (SRS), historically commonly using ^111^In-labeled-DTPA-octreotide (Octreoscan) (20). However, its diagnostic utility is limited by poor image quality, less spatial resolution and a prolonged imaging protocol. More recently somatostatin receptor-based ^68^Gallium positron emission tomography (^68^Ga)-PET/CT imaging has been used due to the higher spatial resolution of PET *vs.* SPECT (3-6 mm *vs.* 10-15mm), allowing greater visualisation of small tumours, coupled with a 10-fold greater affinity of ^68^Ga-DOTA peptides *vs.* ^111^In-Octreotide for SSTRs (21). The higher sensitivity and specificity of ^68^Ga DOTA PET/CT over conventional SRS has been shown previously (21). In a series of 51 patients with a histologically confirmed NET, who had evidence of disease biochemically or on cross-sectional imaging, but with a negative or equivocal ^111^In-DTPA-octreotide scan, ^68^Ga-DOTATATE PET was able to identify disease in 87% of cases, and in many revealed additional lesions, leading to a change in management in 71% of cases (22). This ability to provide information on primary tumour site, to facilitate accurate staging and influence management has meant it is considered the gold-standard functional imaging technique by many.

Several previous studies have determined the impact of ^68^Gallium-DOTA based PET/CT imaging on patient management in individuals with lung carcinoids (23) or gastroenteropancreatic (GEP) NETS, but much less is known about its impact on patient management in patients specifically with panNETs (21, 24). The primary objective of this study was to determine to what extent ^68^Ga-DOTA PET/CT imaging results provide complementary clinical information to cross-sectional imaging (e.g., confirming suspected metastatic disease suspected on CT/MRI or identifying additional sites of local disease or regional/distant metastasis not seen on CT/MRI) and how this information may have led to an intra-or inter-modality change in management in a clinical population with both sporadic and hereditary panNETs.

## Methods

### Inclusion Criteria

**Figure 1:**
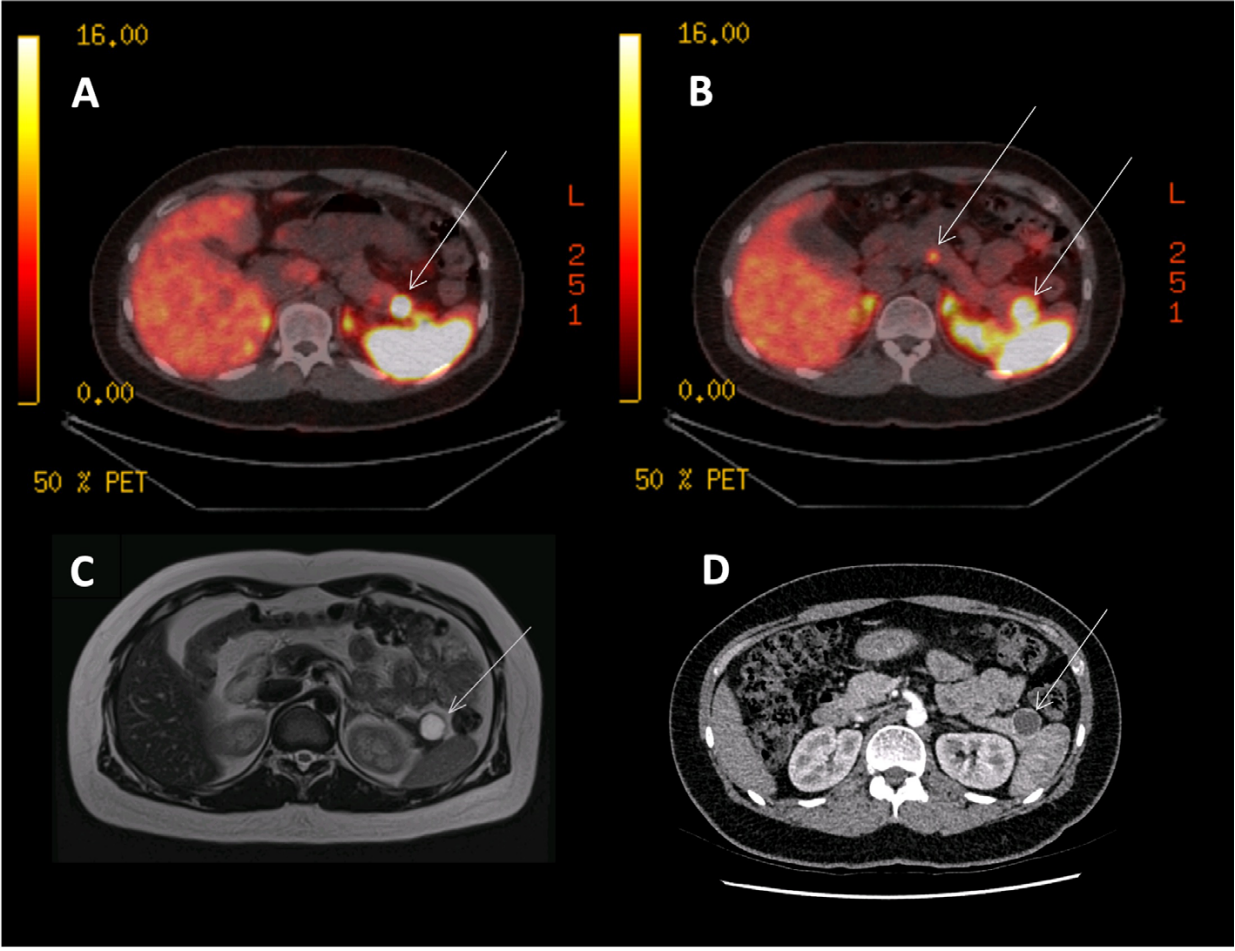
^68^Ga-DOTANOC PET, CT and MRI images of an MEN1 patient with three panNETs (T3N0M0). **(A)** ^68^Ga-DOTANOC PET/CT image showing a single panNET uptaking tracer in the pancreatic tail. **(B)** ^68^Ga-DOTANOC PET/CT image showing two additional PanNETs in the tail and neck of the pancreas. **(C)** MRI scan showing a single lesion in the tail of the pancreas, commented as being ‘cystic’ and needing further imaging and discussion at MDT. **(D)** A CT scan showing a single cystic lesion in the tail of the pancreas, commented on as likely being a cyst.

Patients referred to the NET multi-disciplinary team (NET MDT) meeting at 3 ENETS (European Neuroendocrine Tumour Society) Centres of Excellence (Liverpool, Manchester and London; representing four large hospitals: Aintree University Hospital, Liverpool; Royal Liverpool University Hospital, Liverpool; The Christie NHS Foundation Trust, Manchester; and King’s College Hospital, London) were identified retrospectively from local electronic case-note records. All consecutive patients who underwent a ^68^Ga-DOTA PET/CT scan between July 2015-July 2018 for a clinically suspected or histologically confirmed panNET were identified and included in the analysis (**Figure 1** shows a typical example of imaging findings using complementary cross-sectional and functional imaging). Where multiple/serial ^68^Ga-DOTA PET/CT scans were performed in an individual, each episode was considered and analysed separately as the patient age, disease characteristics, previous treatments and scan findings may/will have been different. ^68^Ga-DOTA PET/CT was used as part of the staging process for all patients with potentially-resectable disease, to detect recurrence in patients who had previous resections and to determine eligibility for PRRT. All patients had undergone comprehensive biochemical testing and cross-sectional imaging with CT/MRI and EUS where appropriate. A data collection template was created based on our related study in lung carcinoids (23).

### Clinical Data

Clinical data were collected retrospectively. Demographic details (age and gender), current status (alive/deceased) and date of death or last follow up were recorded. We included patients with a sporadic panNET or with a familial predisposition to a panNET (multiple endocrine neoplasia type 1 (MEN type 1), von Hippel Lindau (VHL) syndrome, tuberous sclerosis (TSC), neurofibromatosis type 1 (NF1) or miscellaneous others including MUTYH-associated polyposis (MAP) or MAX mutation (familial paraganglioma syndrome)).

### Biochemical Data

All patients underwent biochemical testing for chromogranin A/B and a full fasting gut hormone profile (glucagon, gastrin, somatostatin, pancreatic polypeptide (PP) and vasoactive intestinal polypeptide). The results recorded were those closest to the date of ^68^Ga-DOTA PET/CT scanning, and pre-operatively if the scan was performed pre-operatively. Tumour functionality (functional *vs.* non-functional) was described according to either clinical symptoms (based on a hormonal hypersecretion syndrome) or biochemically (not based on immunohistochemistry analysis).

### Treatment History

Details about treatments previously received were recorded (including disease surveillance). Surgical information included date of surgery, type (Whipple, distal pancreatectomy or enucleation), number of lymph nodes removed, and how many of them were involved with metastatic spread, resection margins (**R0**, cure or complete remission; **R1**, microscopic residual tumour; **R2**, macroscopic residual tumour) and whether there was tumour recurrence following surgery. Systemic therapy was also identified, including use of somatostatin analogues (SSAs), PRRT, molecular targeted therapies and chemotherapy.

### Histopathological Data

Histopathological data was retrieved where tissue samples had been taken (using endoscopic ultrasound-guided fine-needle aspiration (EUS-FNA), fine needle biopsies (FNB) or surgical resection specimens) and undergone histopathological analysis; all histology was reviewed by dedicated experienced NET histopathologists at each centre. Data collected (where available) included the location of the primary pancreatic lesion (unclear, tail, body, head/neck or multifocal) and the number of panNETs.

Pathological data reported included the Ki67 index (%) and mitotic count (counts per High Power Field (HPF)) and the WHO staging (G1, 2 or 3). In some instances, particularly where EUS-FNA is employed, only tumour cytology is possible (to confirm a panNET), and grading (G1, 2 or 3 based on Ki67) is not always possible. This higher diagnostic sensitivity of FNB compared with EUS-FNA has been previously reported (25). Stage at first diagnosis was also recorded incorporating the TNM data measuring the tumour size (T1-T2 *vs.* T3) and determining the presence of lymph node/liver/lung/bone/other metastases.

### Cross-Sectional Imaging Data

All patients underwent cross-sectional imaging with CT scanning of chest, abdomen and pelvis or MRI scanning of the abdomen. Dedicated CT/MRI pancreas specific protocols and endoscopic ultrasound examinations were performed where suggested by the NET MDT.

### ^68^Ga-DOTA PET/CT Imaging Data

All patients underwent functional imaging with a ^68^Ga-DOTA PET/CT scan in line with the local centre-specific imaging protocols acknowledging inherent differences in radiopharmaceutical preparation, scanning equipment and protocols. ^68^Ga-DOTA PET/CT data included the scan date, the specific DOTA-peptide and stage of disease at time of ^68^Ga-DOTA scan (categorised as not present, localised, locally advanced or metastatic). Representative PET/CT images are shown in **Figure 1**.

### Indications for Performing the ^68^Ga-DOTA PET/CT Scan

^68^Ga-DOTA PET/CT scans (in preference to Octreoscan) were performed in all centres on the recommendation of the local MDT according to initial cross-sectional imaging (+/-histological) findings and according to a proposed management strategy decided by MDT consensus. The rationale for performing ^68^Ga-DOTA PET/CT scanning was recorded as one of the following: i) diagnosis and staging for the initial presentation of a panNET ii) post-operative assessment/surveillance/monitoring for relapse; or iii) consideration of PRRT.

### Findings From the ^68^Ga-DOTA PET/CT Scan

Images from all ^68^Ga-DOTA PET/CT images were independently reviewed by the dedicated NET MDT specialist radiologist/nuclear medicine physician (HW, SV, PM) with knowledge of relevant medical information and cross-sectional imaging or EUS findings. Information was collected regarding ^68^Ga-DOTA PET/CT scan findings, including primary tumour (site and size), metastatic spread to the liver, regional lymph nodes, lung, bone and ‘other’ sites and whether the area of uptake was visible on CT, MRI or EUS. In addition, all PET scans were assessed according to whether uptake was equivalent to the background uptake by the liver. Many UK ENETS centres do not routinely report SUV_max_ measurements on ^68^Ga-DOTA PET/CT scans in line with ENETS guidlelines/recommendations.

Findings were categorised as one of the following options: i) provided confirmatory information, concordant with other imaging techniques ii) identified sites of tumour not previously seen with other imaging (i.e., identification of occult metastases), iii) changed options of treatment (surgery/PRRT), iv) ruled out presence of metastases/disease suspected on previous imaging, v) confirmed resection/PRRT response after surgery or PRRT.

### Impact on the Patient’s Clinical Management

Treatment decisions endorsed by the local multi-disciplinary NET team and recorded formally as an MDT outcome were analysed. This was examined by looking at two components. First, we examined the number of episodes where ^68^Ga-DOTA PET/CT provided either confirmatory information in suspected disease or additional information (either by identifying occult sites of disease or excluding suspected sites of disease). Secondly, we assessed the number of episodes where this information assisted or changed management (i.e. by confirmation of eligibility for PRRT or surgery or leading to an inter-modality change in treatment).

### Statistical Analysis

Patient data were anonymised once data collection had been completed, and statistical analysis was carried out using IBM SPSS Statistics for Windows, Version 19.0. Armonk, NY: IBM Corp. (California, USA). Continuous variables were described with median, standard deviation (SD) and range. Categorical variables are presented as frequencies and percentages. Continuous variables were tested for normal distribution by Shapiro-Wilk and Kolmogorov–Smirnov tests. Univariate analysis of categorical variables was performed with chi-square and Fisher’s exact test when appropriate; continuous variables were analysed by Mann-Whitney test as the majority of the continuous variables were not normally distributed. Multivariable analysis consisted of logistic regression for the dichotomous variables and multivariable Mann-Whitney test.

### Ethics

This work was registered as an audit at the lead site of analysis and did not require independent ethical committee approval. Furthermore, all data was completely anonymised and coded as it did not contain any patient-identifiable information it was not necessary to obtain individual patient consent.

## Results

A total of 183 patients with panNETs (97 males and 86 females) were included, for which 224 scans were identified (114 male, 110 female) and analysed. Forty one individuals underwent repeat ^68^Ga-DOTA PET/CT scans (Liverpool Centre) at different points within their treatment pathway (data handling as outlined in Methods).

### Baseline Characteristics

Baseline characteristics of the participants are shown in **Table 1**.

**Table 1:** Summary of patients’ characteristics.

		Whole cohort (n=183)		Sporadic (n=141)		Familial (n=42)	
		n	%	n	%	n	%
**Gender**	Male	97	53	75	53.2	22	52.4
	Female	86	47	66	46.8	20	47.6
**Median age (SD) years**		63 (14.9)	N/A	66 (12.5)	N/A	51 (16.5)	N/A
**Range (upper, lower)**		13,89		13,89		17,79	
**Diagnosis based on**	Imaging	20	11	14	9.9	6	14.3
	Biopsy	163	89	127	90.1	36	85.7
**Ki 67 index (Grade (G))**	G1 (<3%)	72	50.3	58	41.1	14	33.3
	G2 (3-20%)	66		55	39	11	26.2
	G3 (>20%)	5	46.2	4	2.8%	1	2.4
	(range 22-42)	(missing n=40)	3.5	(missing n=24)		(missing n=16)	
**Stage at diagnosis**	Localised	110	60.1	83	58.9	27	64.3
	Locally advanced	26		19	13.5	7	16.7
		43	14.2	37	26.2	6	14.3
	Metastatic	(missing n=4)	23.5	(missing n=2)		(missing n=2)	
**Status**	Alive	163	89.1	124	87.9	39	92.9
	Deceased	17		15	10.6	2	4.8
		(missing n=3)	9.3	(missing n=2)		(missing n=1)	

### Genetic Data

Data for the whole cohort are shown, but additionally we compare data between sporadic panNETs and familial panNETs. Sporadic panNETs arose in 141 patients with panNET-associated familial syndromes in 42 individuals: 36 with MEN1, 2 with VHL, 1 each with TSC, NF1, paraganglioma, MAX mutation and MUTYH-associated polyposis.

### Age

The median age of individuals with familial panNETs was younger than that for those with sporadic panNETs (51 *vs.* 66 years respectively).

### Histopathological Classification

Where available, diagnosis was based on biopsy (either from EUS-FNA, tissue biopsy or from histopathological examination of a resected specimen) for the majority of cases (n=163; 89%); this proportion was similar for sporadic and panNETs (n=127; 90.1% *vs.* n=36, 85.7% respectively). In cases where a tissue biopsy was not performed, diagnosis was based on imaging characteristics. Frequency of tumour grades were G1, 2 and 3: 50.3%, 46.2% and 3.5% respectively for the whole cohort, and the proportions were similar between sporadic and familial panNETs. The reason for the overwhelming predominance of G1 and G2 tumours is likely because our retrospective analysis was based on identifying those patients who had undergone galluim PET/CT scanning. This investigation would usually be reserved for lower grade NETs.

### Extent of Metastatic Spread

Similarly, most tumours were localised (60.1%) or only locally advanced (14.2%) at diagnosis with 23.5% metastatic; familial tumours tended to be more localised than sporadic tumours. The sites of metastatic spread were as expected: liver (60, 32.8% of the entire series), lung (18, 9.8% of the entire series), bone (26, 14.2% of the entire series) and peritoneum (1, 0.5%).

### Functionality of the Tumour

Overall, the majority of tumours were non-functional (73.2%) (**Table 2**). As would be expected, functionality was more common in those with familial panNETs (40.5%) versus those with sporadic panNETs (21.3%).

**Table 2:** Functional hormone secretory patterns.

	Whole cohort (n=183)		Sporadic (n=141)		Familial (n=42)	
	Number, n	%	Number, n	%	Number, n	%
**Functionality**						
Functional	39	21.3	22	15.6	17	40.5
Non-functional	134	73.2	110	78	24	57.1
**Specific functional excess**						
Insulinoma	13	7.1	9	6.4	4	9.5
Gastrinoma/Zollinger-Ellison syndrome	12	6.5	5	3.	4	9.5
Glucagonoma	7	3.8	3	2.1	4	9.5
PTHrP secreting	1	1	2	1.4	–	–
Ectopic ACTH secretion	1	0.5	1	0.7	–	–
Pancreatic polypeptide	1	0.5	–	–	1	2.4
Proinsulin	1	0.5	1	0.7	–	–
VIP and glucagon co-secretion	1	0.5	1	0.7	–	–

Specific functional excess does not include carcinoid syndrome. Missing data not displayed. Percentages are based on the whole sample including missing data in this table. PTHrP, parathyroid hormone-related peptide; ACTH, adrenocorticotrophic hormone; VIP, vasoactive intestinal polypeptide.

### Treatment Characteristics

The majority of patients were treated with curative resection (68%) (**Table 3**). Only a small number received somatostatin analogues, chemotherapy or PRRT.

**Table 3:** Characteristics of tumour staging and treatments. Percentages are calculated on the valid data excluding the missing data.

		Whole cohort (n=183)		Sporadic (n=141)		Familial (n=42)	
		n	%	n	%	n	%
**Curative resection**	Yes	124	67.8	98	69.5	26	61.9
	No	59	32.2	43	30.5	16	38.1
**T pathological stage**	**T1**	55	36.9	41	35.0	14	43.8
**(based on resection specimen)**	**T2**	37	24.8	26	22.2	11	34.4
(Tumour size)
	**T3**	42	28.1	36	30.8	6	18.8
	**T4**	15	10.1	14	12.0	1	3.1
**N pathological stage**	**N0**	96	61.1	76	62.3	20	57.1
**(based on resection specimen)**	**N1**	60	38.2	45	36.9	15	42.9
(Lymph node)	**N2**	1	0.6	1	0.8	0	
**Metastasis M at diagnosis**	**M0**	130	74.7	98	72.6	32	82.1
	**M1**	44	25.3	37	27.4	7	17.9
**Resection margins**	**R0**	76	72.4	64	74.4	12	63.2
	**R1**	25	23.9	20	23.3	5	26.3
	**R2**	4	3.8	2	2.3	2	10.5
**Type of treatment**	**SSA** (Y/N)	39/97	40.2	32	82.1	7	17.9
	**Chemotherapy**	25/116	21.6	23	92.0	2	8.0
	**PRRT** (Y/N)	18/118	15.3	15	83.3	3	16.7

### Indications for Performing ^68^Ga DOTA PET/CT Scans

There were a variety of indications for performing the ^68^Ga-DOTA PET/CT, including diagnosis in cases of suspected panNETs; completing staging in patients with suspected localised panNETs who were being considered for surgical resection (40%), in patients being considered for PRRT (8%), or in post-surgical patients assumed to be disease-free as part of surveillance or monitoring for relapse (53%) (**Table 4**).

**Table 4:** Rationale for performing Ga^68^ DOTA PET/CT scans.

	Whole cohort (n=224)		Sporadic (n=172)		Familial (n=52)	
	Number, n	%	Number, n	%	Number, n	%
**Diagnosis and staging**	88	40	62	36	26	50
**Consideration for PRRT**	17	8	15	9	2	4
**Post-operative assessment/surveillance/monitoring for relapse**	117	53	93	55	24	46
**Missing**	2		2		0	

### The Impact of ^68^Ga DOTA PET/CT Imaging on Patient Management

Findings from the scan were categorised as follows (**Table 5**): in 53.5% of episodes (n=107) the scan findings provided confirmatory evidence available from cross-sectional imaging. In 28.5% of episodes (n=57) the scan identified sites of disease not previously seen with other imaging. There was no evidence that functional imaging provided any advantages over cross-sectional imaging for any specific sites of disease.

**Table 5:** Findings from Ga^68^-DOTA PET/CT imaging and the impact on patient management.

	Whole cohort (n=224)		Sporadic (n=172)		Familial (n=52)	
Findings form the Ga68 DOTA PET/CT imaging	Number, n	Valid %	Number, n	Valid %	Number, n	Valid %
Confirmed information derived from other imaging modalities	107	53.5	81	54.7	26	50.0
Identified sites of cancer not previously seen with other imaging	57	28.5	40	27.0	17	32.7
Inter-modality change of treatment	24	12	17	11.5	7	13.5
Ruled out presence of metastases/disease suspected on previous imaging	10	5	8	5.4	2	3.8
Confirmed resection/PRRT response	2	1	2	1.4	N/A	N/A
Missing	24	N/A	24	N/A		
**Impact on patient management**						
**Additional information provided**						
Yes	119	53.8	88	52.1	31	59.6
No	102	46.2	81	47.9	21	40.4
Missing	3	–	3	–	–	–
**Influenced management**						
Yes	85	39.4	64	39	21	40.4
No	131	60.6	100	61	31	59.6
Missing	8	–	8	–	–	

In 12% of cases (n=24) this led to an inter-modality change in treatment and in 5% (n=10), the scan ruled out the presence of metastases/disease suspected on previous imaging. In 1% (n=2), the scan confirmed adequacy of resection/PRRT response. In 24 episodes these data were missing. In total, new information from the scan was provided in 53.8% of cases (n=119) and influenced management in 39.4% of episodes (n=85).

### Death

Eighty nine percent of patients were still alive with only 9% deceased

Univariate analysis, comparing familial syndrome to sporadic, revealed that there were statistical differences in hormone related syndromes, favouring familial cases (p<0.001). The location of the primary was multifocal more frequently in familial cases (p<0.001) and in sporadic cases, the stage was more commonly metastatic (p=0.009). There was somatostatin analogue use more often in the sporadic cases (p=0.03). Also, there were significant differences in the outcome of the resection in the 2 groups (p=0.025); R0 was more often observed in the sporadic cases. However, no statistical difference was found regarding WHO grade, being predominantly G2 in both groups.

The multivariable analysis (logistic regression) did not show any significant independent variable associated with the change or impact on management.

## Discussion

The findings of this study, conducted in a large cohort of patients with sporadic and familial (predominantly MEN1-related) panNETs highlight the role of ^68^Ga-DOTA PET/CT imaging in the diagnosis, staging and determination of treatment options in patients with panNETs. The scan identified sites of disease that had not been identified with other imaging in 28.5% of patients, excluded suspected disease sites in 5% and confirmed resection/PRRT response in 1% of cases. Overall ^68^Ga-DOTA PET/CT imaging impacted on patient management by providing additional information over cross-sectional imaging in 53.8% of cases and influenced management in 39.4% of cases. Ga^68^-DOTA PET/CT imaging may aid or change management by identification of additional sites of pancreatic or metastatic disease not observed using CT/MRI, or by modifying the intended surgical approach or leading to substitution of surgery for systemic therapy where unresectable disease is demonstrated. Similarly, ^68^Ga-DOTA PET/CT was necessary prior to PRRT treatment to demonstrate eligibility by confirming avidity of the tumour to SSTR-based tracers (26).

Our findings are consistent with those of other studies that have examined the role of functional imaging in patients with panNETs. The choice of functional imaging modality depends on the biological characteristics of the NETs, where histological grade is available: ^18^F-FDG PET/CT is preferred for more aggressive, higher grade and less well differentiated neuroendocrine neoplasms while ^68^Ga-DOTA PET/CT was found to be superior to ^18^F-FDG PET/CT in patients with low-grade tumours. In general, in patients with a NET with a lower Ki67 index, the use of ^18^F-FDG PET/CT should be limited, while for higher G2 and G3 NETs, combined ^68^Ga-DOTATATE and ^18^F-FDG PET/CT can be considered (27). In the current study, we do not report findings of the ^18^F-FDG PET/CT scans (if/where they were undertaken) in those patients with higher G2 and G3 NETs as this was not the focus of the research.

The role of ^68^Ga-DOTA PET/CT imaging in patients with NETs has generally been well established (28, 29) and it is now acknowledged as the functional imaging modality of choice in the current standards of care, where available (26, 30). Although the potential utility of SRS is acknowledged in the diagnosis of panNETs, ^68^Ga DOTA PET/CT is generally considered superior to conventional SRS. As no SRS was undertaken in this cohort, no comparative data between SRS and PET/CT is available. However, much of the previous analysis relating to ^68^Ga-DOTA PET/CT imaging in patients with NETs has related to heterogeneous populations of gastroenteropancreatic (GEP) NETs, with far fewer studies evaluating the utility in panNETs exclusively (31–33). Reassuringly, a recent systematic review and meta-analysis of 14 studies including a total of 1,561 patients demonstrated that somatostatin receptor directed PET-CT effected a change in management in 44% (range 16-81%) consistent with our findings (34).

Furthermore, in the studies examining panNETs exclusively, most reflect analysis in sporadic panNETS, and few have included MEN1-related panNETs specifically (**Supplementary Table 1**). The strength of this study is that it includes a large cohort of patients with MEN1-associated panNETs. Current clinical practice guidelines for the diagnosis and surveillance of non-functional panNETs suggest the use of biomarkers (such as chromogranin A), but the results of recent biomarker studies highlight a low diagnostic accuracy for MEN1-associated panNETs (35, 36). The MEN guidelines also recommend anatomical imaging modalities such as CT/MRI and EUS without mentioning these newer functional imaging modalities (17). The inclusion of the genetic cohort (who are under regular active clinical, biochemical and radiological surveillance) may explain the proportion of patients with localised disease permitting complete resection. There have been only a small number of both retrospective and prospective analyses of the role of ^68^Ga DOTA PET/CT specifically considering MEN1-associated panNETs (21, 24, 37, 38). Applied to MEN1 specifically, ^68^Ga DOTA PET/CT was more sensitive than Octreoscan or CT scan in a study of 26 cases comparing multiple imaging modalities (including ^111^I-pentetreotide SPECT-CT and triple phase CT); in 38.5% of patients ^68^Ga DOTA PET/CT detected additional metastases (24). In 31%, the addition of ^68^Ga DOTA PET/CT scan findings caused a change in management recommendations (related to detection of metastases). The group recommended that this imaging modality should be integrated into screening and surveillance of panNETs in MEN1. In a larger cohort of 131 patients with GEP-NETS studied prospectively (of which a proportion were panNETs), ^68^Ga DOTATE PET/CT was demonstrated to be significantly superior to CT and ^111^In-pentetreotide (demonstrating 95.1, 45.3 and 30.9% of lesions respectively) (21). However, we would argue that functional imaging is only advocated in cases where surgical resection or PRRT is being considered or in general where the results may influence management.

A recent analysis of 5287 cases of panNET in the Surveillance, Epidemiology and End Results Program (SEER) US national database, demonstrated that the median survival from diagnosis was 4.1 years (95% CI 3.9-4.4), but this varied dramatically according to patient characteristics. Functioning (*vs.* non-functioning) panNETs, younger age at diagnosis, localised disease, lower tumour grade and surgical treatment were all associated with lower mortality. However, even the most favourable combination of risk factors were still associated with some reduction in normal life expectancy (16) and so there is a significant need for early detection and selection of the most appropriate treatment to improve patient outcomes. The optimal management of patients with panNETs depends on their size, functionality, tumour grade and stage. The decision as to whether management should consist of ongoing surveillance, surgical resection, medical or systemic therapy requires a multidisciplinary approach. The positive impact of surgical resection on survival in patients with nonfunctioning panNETs has been repeatedly demonstrated (39, 40). Current recommendations for surgical intervention suggest that resection should be considered for any functional panNET or for non-functional panNETs >2cm (41). Small nonfunctioning panNETs are often indolent neoplasms without lymph node metastasis. However, within the context of MEN1, analysis of the literature for MEN1-related panNETs highlighted a very low growth rate of small non-functioning (NF) panNETs suggesting a need for a re-evaluation of the timing and frequency of surveillance and optimal treatment approach (42).

Our results showed a lower percentage of R0 resection in familial cases. This could be related to a more conservative surgical approach. However, in our series, it could be partially explained by a higher number of missing data within this smaller group (the familial cases).

Limitations of this study are acknowledged. First, we acknowledge that this was a retrospective data collection with the inherent limitations (e.g., less comprehensive and a lack of standardisation of data collection) and secondly that we were also unable to record detailed justification of the management decisions other than *via* the written NET MDT outcomes. Histopathological data was available for the vast majority (89%) of patients but not for the entire cohort. This reflects real-world management where tissue sampling may not be feasible or clinically appropriate. Furthermore, we accept PET/CT scanning equipment and scanning protocols (including use of slightly different radiolabelled somatostatin analogues (DOTA-NOC *vs.* TOC *vs.* TATE) are not perfectly aligned between centres. We do not routinely quantify tracer uptake on the PET/CT scans e.g., using SUV_max_ or Krenning score however this may be a more relevant consideration to assess treatment response rather than initial treatment choice. Finally, we do not have simultaneous Octreoscans to highlight the superior sensitivity of somatostatin receptor-directed PET-CT over octreoscans. However, the significant strengths of the current study include the large size of the series including both sporadic and familial, inclusion of three ENETS centre of excellence and extensive multi-disciplinary involvement including 4 experienced nuclear medicine PET/CT physicians.

Thus, although we cannot comment on the role of functional imaging (*versus* cross-sectional or biochemical surveillance) as part of a surveillance protocol in patients who have received treatment for their NET, we have shown that ^68^Ga-DOTA PET/CT has a significant complementary role in staging and guiding treatment in patients with sporadic and familial panNETs with newly diagnosed or recurrent disease, particularly where surgical resection or treatment with PRRT is considered.

## Data Availability Statement

The raw data supporting the conclusions of this article will be made available by the authors, without undue reservation.

## Ethics Statement

Ethical review and approval was not required for the study on human participants in accordance with the local legislation and institutional requirements. Written informed consent for participation was not required for this study in accordance with the national legislation and the institutional requirements.

## Author Contributions

All authors contributed to data collection, drafting and editing the manuscript. Statistical analysis was undertaken by JB. All authors contributed to the article and approved the submitted version.

## Conflict of Interest

The authors declare that the research was conducted in the absence of any commercial or financial relationships that could be construed as a potential conflict of interest.
