# Supplementary material for: The Impact of 68Gallium DOTA PET/CT in Managing Patients With Sporadic and Familial Pancreatic Neuroendocrine Tumours
Source: Front Endocrinol (Lausanne). 2021 Jun 7;12:654975. doi: 10.3389/fendo.2021.654975 (PMC8215358; doi:10.3389/fendo.2021.654975)
Supplement: Supplementary file 1 [file Table_1.docx]

**Supplemental Table 1** Familial panNETs and the role of Ga68 DOTA-peptide PET/CT (**PS**=prospective study, **RS**=retrospective study)

**MEN1**

| **Study Type** | **Gallium Results** | | | | |  |
| --- | --- | --- | --- | --- | --- | --- |
| **Study/study type** | **Group size**  **and age (y)** | **Primary Outcome measure** | **Identified new sites of disease** | **How many added extra information?** | **How many changed management?** | **Conclusion** |
| *Froeling et al. (2012)(Froeling et al., 2012)*  **RS** | **MEN1 group only**  **Number:**21 (F:10, M:11) (28 scans)  **Age:**41.3 (16-78) y | Using records of NET MDT, to assess impact on diagnosis and therapeutic management of Gallium DOTATOC. |  |  | 10/21 (47.6%)  (n=9 indicated surgery, n=1 altered surgical approach) | Substantial (21 patients) MEN1 study showing a high detection rate of NETs in MEN-1. |
| *Sadowski et al. (2015)(Sadowski et al., 2015)*  **PS** | **MEN 1 only**  **Number:**26 (F:9, M:17)  **Age:**42 (19-82) y  **Tumour type**  NF-panNET 7  Gastrinoma 3  Insulinoma 3  Thymic NET 1 | Comparison of the accuracy of imaging with Ga DOTATATE, In-pentreotide and CT with clinical, biochemical and pathology data | ^68^Ga detected 107 lesions  ^111^In detected 33 lesions  CT detected 48 lesions | 10/26 patients (38.5%) additional metastases were detected | 8/26 (**31%)** change of management | ^68^Gallium-DOTATATE PET/CT is more sensitive than In-pentetreotide SPECT/CT and CT in MEN1 patients.  Suggests integration into screening and surveillance programmes. |
| *Sadowski et al. (2016)(Sadowski et al., 2016)*  **PS** | **Large sporadic and MEN1 group (size of MEN cohort not specified)**  **Number:**131 (F:74, M:57)  **Age:**51(19-82) y | **Detection rate of lesions by modality:** ^68^Ga-DOTATATE *vs.*^111^In-pentetreotide *vs.* CT and MRI scanning. | 95.1% lesions detected by ^68^Ga  45.3% anatomic lesions detected by CT/MRI  30.9% lesions with^111^In | 93/131 (71%) additional lesions than ^111^In-pentetreotide SPECT/CT.  69 /131 (52.7%) additional lesions than CT. | 43/131(32.8%)  change of management | ^8^Gallium-DOTATATE imaging provides important information for accurate staging of GEPNETs, even in the absence of biochemical evidence of disease. |
| *Albers et al. 2017 (Albers et al., 2017)*  **PS** | **Number:**33 (17M, 16F)  **Age:**44 (19-74)  **Tumour type**  2 insulinoma  6 ZES  31 NF-panNETs | ^68^Ga-DOTATATE *vs.* CT and MRI scanning. | ^68^Ga DOTATOC PET/CT detected 55 NETs in 23/33 patients  Conventional imaging 145 NETs in 31/33 patients | 90 NETs detected by CT/MRI missed by ^68^Ga PET/CT | Detected more liver and LN metastases | Gallium detected more liver and lymph metastases in patients with known metastatic disease **BUT** failed to change management. |
